# Phytohormones regulate the abiotic stress: An overview of physiological, biochemical, and molecular responses in horticultural crops

**DOI:** 10.3389/fpls.2022.1095363

**Published:** 2023-01-06

**Authors:** Yi Zheng, Xiaonan Wang, Xin Cui, Kefeng Wang, Yong Wang, Yuhui He

**Affiliations:** ^1^ School of Life Science, Changchun SCI-TECH University, Changchun, Jilin, China; ^2^ School of Architecture and Urban Planning, Changchun University of Architecture and Civil Engineering, Changchun, Jilin, China

**Keywords:** environmental stresses, horticultural plant growth, plant hormones, plant physiology, photosynthesis

## Abstract

Recent changing patterns of global climate have turned out to be a severe hazard to the horticulture crops production. A wide range of biotic and abiotic stresses often affect plants due to their sessile nature. Horticultural crop losses are mainly caused by abiotic factors such as drought, salt, heat, cold, floods, and ultraviolet radiation. For coping up with these adversities, well-developed mechanisms have been evolved in plants, which play a role in perceiving stress signals and enabling optimal growth responses. Interestingly, the use of phytohormones for suppressing the impact of abiotic stress has gained much attention in recent decades. For circumvention of stress at various levels, including physiological, molecular, as well as biochemical, a sophisticated mechanism is reported to be provided by the phytohormones, thus labeling these phytohormones a significant role in plant growth and development. Phytohormones can improves tolerance against abiotic stresses by increasing seed germination, seedling growth, leaf photosynthesis, root growth, and antioxidant enzymes and reducing the accumulation of reactive oxygen species, malonaldehyde, and electrolyte leakage. Recent discoveries highlight the significant role of a variety of phytohormones including melatonin (MEL), Gamma-aminobutyric acid (GABA), jasmonic acid (JA), salicylic acid (SA), brassinosteroids (BRs), and strigolactones (SLs) in abiotic stress tolerance enhancement of horticultural plants. Thus, current review is aimed to summarize the developmental concepts regarding role of phytohormones in abiotic-stress mitigation, mainly in horticultural crops, along with the description of recent studies which identified the role of different phytohormones in stressed environments. Hence, such a review will help in paving the path for sustainable agriculture growth *via* involvement of phytohormones in enhancement of abiotic stress tolerance of horticultural crops.

## Introduction

**Figure 1:**
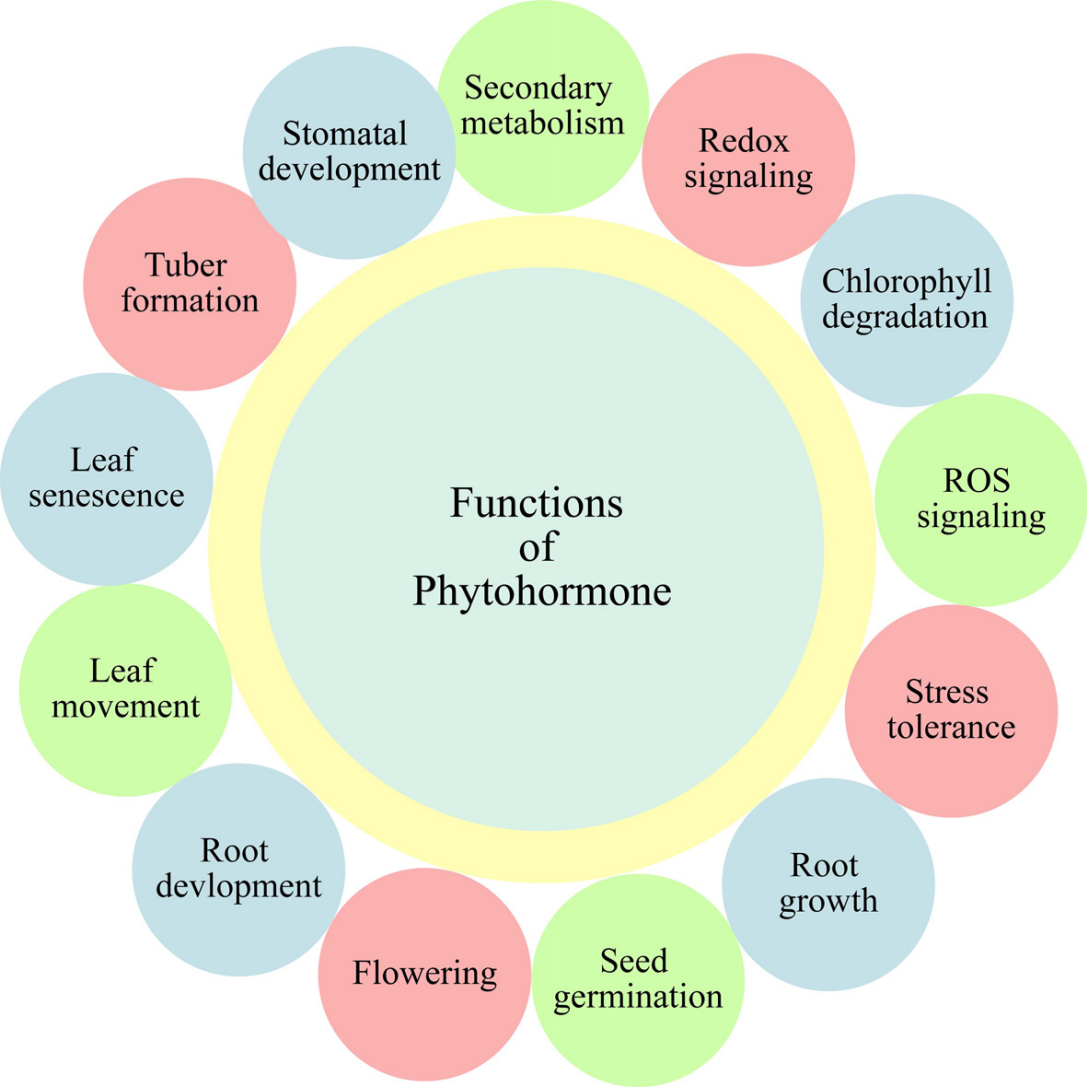
Different functions of phytohormones in horticultural plants.

With recent changes in global climate, along with the elevations in world population, an increment in the agricultural productivity is a need of time. The estimated agricultural output by the mid-century must be 70 percent more than the current output, for fulfilling the requirements of world growing population (Francini and Sebastiani, 2019). Moreover, climate variabilities also significantly affect production of horticultural crops. Various abiotic and biotic stresses are important factors, limiting the agricultural yield and productivity (Mangal et al., 2022). The response of plants towards varying environmental stimuli is one of the most critical questions, both for agronomists and plant biologists. Amid different environmental stresses affecting growth and development of plants, salt, drought and heat stress are the most important, as well as common ones (Wani et al., 2016). Due to the complex characteristics associated with stress tolerance, traditional breeding techniques show low efficacy, thus needing advancements for filling the gap between world food supply and demand. Development of new and effective methods is a necessity in this area. One of the viable alternatives and realistic option for growing highly productive climate-resilient crops is the phytohormones. Recently, phytohormones are emerged as highly eco-friendly alternative approach, which help to enhance abiotic stress tolerance, particularly in horticultural plants. Phytohormones are the plants’ released chemical regulators targeting the regulation of plant responses, growth, and development under environmental stresses (Kohli et al., 2013; Verma et al., 2016). The important role of phytohormones under abiotic stresses is through coordination of differential signal transduction pathways (Saini et al., 2021; Salvi et al., 2021). They also get involve in regulation of different stimuli, both internal and external, thus bringing key changes in development of plants (Ku et al., 2018). Hitherto, the role of phytohormones as signaling molecules in abiotic stress resistance has been studied in horticultural plants (Wu et al., 2018; Sytar et al., 2019), and these phytohormones also play significant role in crop production of horticultural plants (**Figure 1**) (Ciura and Kruk, 2018). In recent studies on abiotic stress tolerance, the regulatory role of phytohormones in various plant processes including physiological, molecular, and biochemical, has been highlighted (Arif et al., 2020). In the light of above background, the key focus of current review is to highlight the conceptual improvements in abiotic stress tolerance of horticultural plants through different phytohormones’ functioning, including brassinosteroids (BRs), melatonin (MEL), salicylic acid (SA), jasmonates (JAs), strigolactones (SLs), and gamma-aminobutyric acid (GABA). **Figure 1** depicts the illustration of role of these phytohormones.

## Effects of abiotic stress on horticultural crop performance

**Figure 2:**
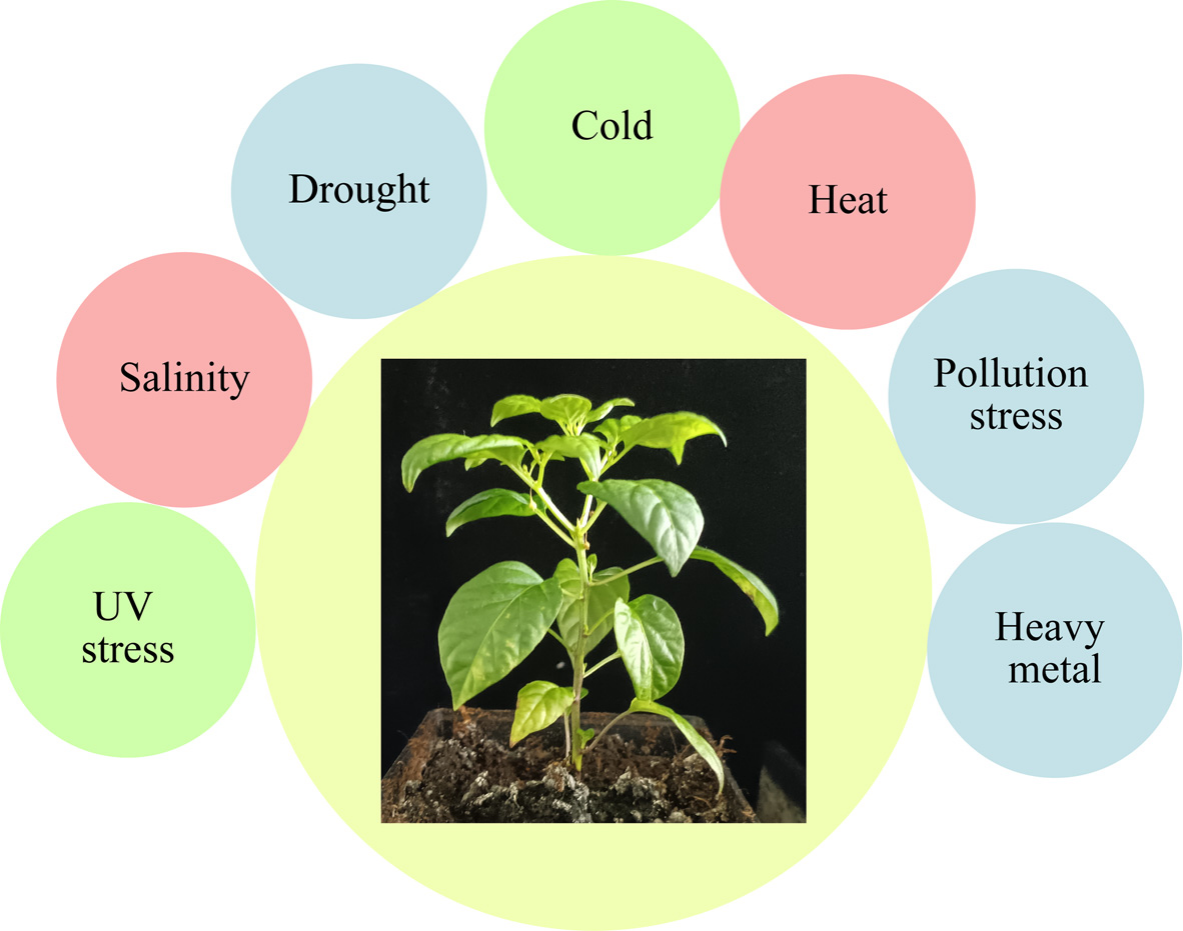
Illustration of several stress factors that impede horticulture plants growth and development.

Starting from seed germination and through the whole life cycles, plants face multiple stresses (**Figure 2**). Various abiotic stressors cause crop losses *via* affecting quality and yield of crop products, including salinity, heat, drought, and nutrient deficiency stresses (Andreotti, 2020). Abiotic stresses impact is not only on yield but also on the product quality, causing morphological, physiological, and biochemical alterations (Rao et al., 2016). Recent changing climatic events also pose multiple abiotic stresses to horticultural crops. Changing climate is also labeled as an eminent challenge that agriculture sector must suffer in the future (Francini and Sebastiani, 2019; Shahid et al., 2021; Gao et al., 2022). A wide range of stress responses are reported in plants, including decline in photosynthetic machinery yield, leaf water potential, membrane integrity, photosynthetic pigments, plant growth, and yield (Ullah et al., 2018). Further, single and/or multiple stress conditions affecting 90 percent of agricultural lands. The horticulture sector is therefore actively seeking for new agronomic tools that are able to contrast the adversities of environmental factors, while maintaining the overall sustainability as well as quality of the production. In this regard, the horticultural plants are protected through various plant hormones against abiotic stressors (**Table 1**).

**Table 1:** Phytohormone-mediated regulation of abiotic stress-impacts in horticultural crops: a summary of representative research.

Pant hormone	Specie	Stress type	Key findings	Reference
Brassinosteroids	Tomato	Salinity	I, II, III, IV, X	Ahanger et al., 2020
Salicylic acid	Colver	Aluminum	I, IV, V, VI,	Bortolin et al., 2020
Jasmonic acid	Citrus	Cold	IV, V, VI,	Habibi et al., 2019
Melatonin	Tomato	Acid rain	I, II, IV, V, VIII	Debnath et al., 2018
Strigolactones	Pea	Cold	I, III, VIII	Cooper et al., 2018
GABA	Melon	Saline-alkaline	I, IV, V	Xiang et al., 2016
Salicylic acid	Eggplant	Cold	IV, V, VIII,	Chen et al., 2011
Jasmonic acid	Pepper	Waterlogging	III, V, VI, VIII,	Ouli-Jun et al., 2017
Melatonin	Apple	Drought	I, II, VIII, XI	Liang et al., 2018
GABA	Peach	Cold	IV, V, V,	Yang et al., 2011
Brassinosteroids	Tomato	Nickel	I, II, V, VI, IX	Nazir et al., 2019
Salicylic acid	Peppermint	Cadmium	I, II, VIII, IX,	Ahmad et al., 2018
Strigolactones	Tomato	Drought	I, II, III,	Visentin et al., 2016
Jasmonic acid	Pea	Heat	I, IV, V,	Shahzad et al., 2015
Melatonin	Loquat	Drought	III, IV,	Wang et al., 2021
Salicylic acid	Okra	Cold	IV, V	Bahadoori et al., 2016
Strigolactones	Grapevine	drought	I, II, III, IV, V,	Min et al., 2019
Melatonin	Watermelon	Vanadium	II, III, IV, V, IX	Nawaz et al., 2018
GABA	Tomato	Cold	I, VI, V	Malekzadeh et al., 2014
Brassinosteroids	Pepper	Cold	II, V, VII, VIII	Yang et al., 2019
GABA	Muskmelon	Calcium nitrate	IV, V, IX,	Hu et al., 2015
Melatonin	Kiwifruit	Drought	I, II, VII, VIII,	Liang et al., 2019
Brassinosteroids	Eggplant	Heat	II, IV, VIII	Wu et al., 2014
Salicylic acid	Tomato	heat	I, IV, V, VI,	Singh and Singh, 2016
GABA	Pepper	Low light	II, IV, V, VIII	Li et al., 2017
Strigolactones	lettuce	Drought	I, II, III, V, VIII,	Ruiz-Lozano et al., 2016
Brassinosteroids	cucumber	Cadmium	II, IV, V, VIII, IX	Shah et al., 2019
Jasmonic acid	Nightshade	Cadmium	I, VII, VI, IX	Yan et al., 2015
Salicylic acid	Cucumber	Manganese	III, IV, V	Shi and Zhu, 2008

I, growth increased; II, enhanced leaf gas exchange traits; III, root growth improved; IV, reduced oxidative stress level; V, antioxidant enzymes increased; VI, osmolytes content improved; VII, Increased seedling growth; VIII, Pigments content; IX, reduced metal uptake; X, decreased sodium and potassium content; XI, nutrient uptake.

## Phytohormones: Key mediators of plant responses to abiotic stresses

**Figure 3:**
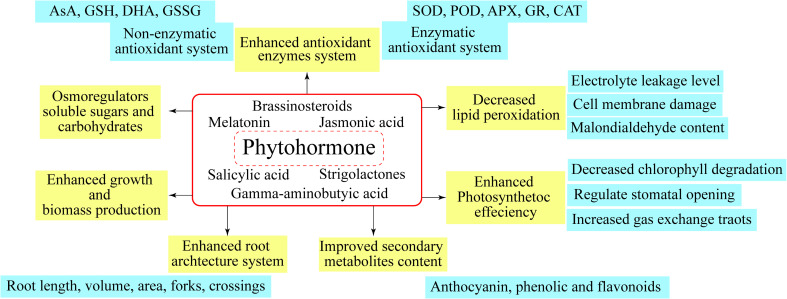
Different plant mechanisms regulated by Phytohormones.

Other than playing roles in developmental processes, endogenous phytohormones are significantly involved in abiotic stress tolerance, and are mentioned as key mediators of responses in plants under stress conditions. Plant hormones are small and signaling molecules, acting virtually to some extent, in every aspect of growth and development of plants (Ullah et al., 2018). The acting mechanism behind various processes can vary with different hormones. Thus, a single hormone is sometimes observed to regulate a wide range of processes, both cellular and developmental, whereas, simultaneously, a single process might get regulated by multiple hormones (Ciura and Kruk, 2018). Phytohormones which are crucial for growth and development of plants include BRs, MEL, SA, JA, GABA, and SLs (**Figure 3**), providing support and management to plants against biotic and abiotic stressors (Sytar et al., 2019). Hence, the application of phytohormones is carried out for enhancing the future crop stress management research.

## Brassinosteroids

Brassinosteroids (BRs) are steroidal plant compounds that are non-toxic, multifunctional, and poly-hydroxylated (Yasir and Wasaya, 2021). 28-homobrassinolide (28-HBL), Brassinolide (BL) and 24-epibrassinolide (24-EBL) are the most effective BRs which are commonly used in plant molecular and physiological research. Seed germination, cell division, senescence, stomatal opening, seedling development, root growth, and vascular differentiation are all aided by BRs (Ullah et al., 2018; Chaudhuri et al., 2022). Different morphological and physiological processes in horticultural crops are effectively controlled by BRs (Mumtaz et al., 2022). Currently, it is clarified that BRs may confer significant tolerance in plants towards many abiotic stressors such as salt, water logging, drought, metals, and high and low temperatures (Ahammed et al., 2020; Kour et al., 2021). The stress protection and growth regulatory ability of BRs nexuses strongly with the protein and nucleic acid biosynthesis, and photosynthesis related metabolic processes (Upreti and Sharma, 2016). Ding et al. (2012) reported that BRs supplementation significantly enhanced growth and antioxidant enzymes pool and reduced electrolyte leakage (EL), malonaldehyde (MDA), sodium and chloride content in eggplant under salt stress. Under salt stress, BRs application efficiently improved strawberry growth characteristics, ion homeostasis, activity of antioxidant enzymes, leaf photosynthesis, and cellular membrane integrity (Karlidag et al., 2011). Furthermore, 24-EBL treatment increased photosynthesis-related characteristics in pepper under drought stress (Hu et al., 2013). Mahesh et al. (2013) discovered that 24-EBL and 28-HBL supplementation boosted osmolyte content, antioxidant enzymes, and reduced levels of MDA under drought stress in radish crop. Kapoor et al. (2014) revealed that 24-EBL application enhanced radish photosynthetic pigments content, level of secondary metabolites under metal (cadmium and mercury) toxicity. Moreover, 24-EBL significantly modulates the ascorbate-glutathione (AsA-GSH) pool.

Exogenous BRs have been proven to alleviate the deleterious effects of abiotic stress on a number of horticultural plants. In *Cucumis sativus* L., *Solanum lycopersicum* L., *Prunus persica* L., and *Raphanus sativus* L., BRs boosted antioxidant enzymes (SOD, CAT, POD, APX), chlorophyll content, secondary metabolites, and gas exchange components (Ge et al., 2014; Choudhary et al., 2012). Furthermore, the use of 24-EBR significantly enhanced secondary metabolite, leaf photosynthetic parameters, and chlorophyll content in cucumber (Yu et al., 2004). Choudhary et al. (2011) discovered that supplementation with 24-EBR effectively improved growth parameters, secondary metabolites, antioxidant enzyme activity, and decreased oxidative stress biomarkers in *Raphanus sativus* L. under copper stress. Under metal toxicity, 24-EBL efficiently enhanced root architectural system, chlorophyll content, antioxidant metabolites, and AsA-GSH pool and lowered oxidative damage in grape (Zhou et al., 2018). Under nickel toxicity, 24-EPR supplementation effectively improved metabolic function of tomato (Soares et al., 2016). Under cadmium stress, Hayat et al. (2012) discovered that BRs treatment boosted growth status, yield attributes, photosynthetic pigments content, and antioxidant system in *Solanum lycopersicum*. In another study, when grapevine seedlings are exposed to chilling stress, 24-EBL application balances the antioxidant machinery, and improves osmolyte content (Xi et al., 2013). The positive regulation of growth and development of sweet pepper plants by BR application has been reported through suppressing the adversities of different abiotic stress factors. (Hegazi et al., 2017), pea (Shahid et al., 2011), cucumber (Hu et al., 2010), and fenugreek (Swamy et al., 2014), and radish (Alper and Sharma, 2013) by overcoming the negative effect of multiple abiotic stresses. Though the variability in BRs’ physiological responses is due to complex actions of molecular mechanisms, the plants’ stress response management is highly dependent on their potential of improving tolerance towards abiotic stresses through BRs. Deep investigations are required in future for effective gains and enhanced scope of BRs research, mainly through studying the BRs stress tolerance mechanisms.

## Melatonin

Melatonin is a potential antioxidant with a low molecular weight (Nawaz et al., 2016). Previous research demonstrated that MEL is involved in a variety of plant activities during the vegetative and reproductive stages of plants, including seed germination, seedling growth, root architecture, blooming, plant growth, and leaf senescence (Jahan et al., 2021a; Wu et al., 2021). It has been reported hitherto that plant growth status is maintained normally due to positive effects of MEL under abiotic stresses (Tiwari et al., 2020; Heshmati et al., 2021). The molecule was detected in horticultural plants in 1993, in morning glory and tomato, according to Van Tassel (1997). In plants most common way, MEL controls stress *via* increasing the antioxidative defense mechanisms, as well as reactive oxygen species (ROS) scavenging. Nawaz et al. (2018) revealed the surprisingly increased growth traits, activity of antioxidant enzymes, root morphology, chlorophyll content, and suppressed oxidative damage in MEL-pretreated watermelon seedlings in vanadium toxicity. Foliar application of melatonin dramatically reduced ROS-induced damage and enhanced growth, metabolites level, and activity of antioxidant enzymes in strawberry seedling under cadmium stress (Wu et al., 2021). Sharma and Zheng (2019) revealed the protective role of MEL in horticultural plants through prevention of damages to antioxidative defense system, photosynthetic apparatus, by regulation of oxidative stress and other defense mechanisms. Various positive effects of exogenously applied MEL are present in literature, including prevention of photosynthetic damages to chloroplast, and improvement of intact spongy tissues, leaves water content, and turgor pressures under drought stress (Tiwari et al., 2020).

The ROS scavenging efficiency gets enhanced and antioxidant defense system of plants gets triggered by the application of MEL. In horticultural plants, the related phenomenal mechanisms for scavenging of H_2_O_2_ and regulating superoxide anions are well studied in the form of ascorbate-glutathione cycle’ regulation (Vielma et al., 2014; Ahammed et al., 2019). The photosynthetic damage induced by abiotic stress has been minimized with exogenous treatment of MEL in tomato (Jahan et al., 2021b), watermelon (Li et al., 2017a), peach (Cao et al., 2018), cucumber (Zhang et al., 2020), and pepper (Korkmaz et al., 2017). Furthermore, in *Malus hupehensis*, the inhibitory effect of UV-B radiation efficiently mitigated by MEL (1 µM) supplementation. MEL controlled photosynthetic properties and decreased damages to leaf membrane. Furthermore, MEL treatment resulted in a considerable decrease in H_2_O_2_ levels as well as increased antioxidant machinery (Wei et al., 2019). MEL application significantly improved growth status, photosynthesis, anti-oxidant activity of enzymes, and depletion of ROS generation in ozone-stressed grape leaves (Liu et al., 2021). Jahan et al. (2020) observed that MEL application significantly increased micro and macro-nutrient absorption under nickel toxicity in tomato roots and leaves, whereas MEL treatment significantly lowered nickel deposition in both root and shoot system. Exogenous MEL application significantly reduced ROS production and chlorophyll degradation in leafy vegetables like fenugreek, whereas MEL application significantly enhanced antioxidant enzymes, proline content, and photosynthetic pigments under drought condition (Zamani et al., 2019). Additionally, in pepper plants, the H_2_O_2_, and MDA contents and EL level were reduced, and antioxidant enzymes, leaf mineral content, gas exchange elements, leaf area, and seedling biomass were enhanced by the application of MEL under cold stress (Korkmaz et al., 2021). These results suggest the signaling function of MEL, which help to enhance growth and defense mechanisms of horticultural plants under abiotic stresses.

## Salicylic acid

Salicylic acid is a versatile natural phenolic compound and an important signaling molecule (Prakash et al., 2021). SA has been demonstrated to be important in the regulation of plant physiological processes such as seedling growth, root growth, leaf photosynthesis, ion homeostasis, secondary metabolite production, fruit ripening, and antioxidant enzymes system (Hernández et al., 2017; Rajeshwari and Bhuvaneshwari, 2017). Miao et al. (2020) revealed that SA supplementation considerably enhanced cucumbers growth characteristics, photosynthetic capability, and root architecture system under salt stress. Under high temperature stress, SA application considerably boosted leaf water potential, metabolites, leaf gas exchange components, antioxidant enzyme system, and decreased ROS-induced damage in *Solanum lycopersicum* L. (Jahan et al., 2019). SA significantly improved potato growth status, antioxidant enzyme activity, proline content, chlorophyll content, and decreased oxidative damage and reduced cadmium accumulation under cadmium toxicity (Li et al., 2019). Hormonal priming with SA in cucumber seed improves germination of seeds, development of seedling and yield of crop (Rehman et al., 2011). Under water scarcity environments, SA treatment enhanced plant fresh weight, leaf water potential, photosynthetic apparatus, antioxidant enzymes system, anatomical response, and decreased cell damage in tomato (Lobato et al., 2021). Under heat stress, SA increased thermotolerance, chlorophyll concentration, leaf water content, and antioxidant enzyme activity in pepper (Zhang et al., 2019). Moreover, SA improved the photosynthesis of pepper (Kaya, 2021), peach (Zhao et al., 2021), cucumber (Shi et al., 2006), and melon (Zhang et al., 2015). According to Chen et al. (2011), SA application can prevent cold stress-induced oxidative damage in eggplant seedlings by enhancing the activity of antioxidant enzyme and upregulating gene expression. Under high temperature conditions, SA supplementation lowered H_2_O_2_ concentration and controlled the system of antioxidant enzymes in banana plants (Kang et al., 2003). Plants subjected to ozone stress, SA treatment significantly increased seed germination, nitrogen absorption, and root properties (Santisree et al., 2020). Spraying of SA improved antioxidant enzyme activity in pepper leaves subjected to UV-B exposure (Mahdavian et al., 2008).

The extant literature highlights the mitigating effects of exogenous SA in horticultural crops under abiotic stress, including spinach (Shin et al., 2018), tobacco (Dat et al., 2000), rosemary (El-Esawi et al., 2017), pea (Embiale et al., 2016), and strawberry (Ergin et al., 2016). The most prevalent plant responses mediated by SA in horticultural plants are enhanced chlorophyll content, secondary metabolites, proline level, and antioxidant enzyme activity. Conversely, other plant responses to abiotic stress conditions include reduced oxidative damage (strawberry, tobacco, and rosemary) and improved growth characteristics, and yield. The synergistic and antagonistic interactions of SA with nutrients, under both favorable and stressed environments, helps to modulate the growth and development of plants. For example, exogenous application of SA significantly reduced salt (Na^+^) absorption while increasing absorption of mineral nutrients under salt stress in cucumber (Yildirim et al., 2008). Significant increments in antioxidant enzymes (AsA-GSH pathway) and reductions in oxidative damage, along with relevant gene expressions, are exhibited in eggplant seedlings under cold stress (Chen et al., 2011). SA application successfully decreased oxidative damage and boosted antioxidant defense system in okra during cold stress (Bahadoori et al., 2016). SA has the ability to mitigate the harmful environmental effects on horticultural crops.

## Jasmonates

Jasmonic acid (JA) and methyl jasmonate (MeJA) belong to a group of multifunctional compounds called Jasmonates (JAs) (Ullah et al., 2018). They are key plant signaling molecules that regulate plant responses to environmental stress and play a variety of role in plants growth and development (Eyidogan et al., 2012). Furthermore, Photosynthesis, root elongation, stomatal development, leaf senescence, chlorophyll breakdown, and nutritional balance are all regulated by JA (Siva et al., 2015). The crucial role of JA in stress tolerance and adaptability of plants is well documented. Interestingly, the resistance of plants towards environmental stress factors in increased by JA (**Tables 1**, **2**). Exogenous MeJA increased cold stress tolerance in peaches *via* ROS-mediated oxidative damage maintenance and enhanced antioxidant defense mechanism (Jin et al., 2009). Under heat stress, JA application increased antioxidant defense system of grapes seedling (Chen et al., 2006). Furthermore, JA significantly enhanced sugar beet production, antioxidant enzyme activity, and water scarcity tolerance (Ghafari et al., 2020). According to Sayyari et al. (2011), MeJA supplementation significantly increased secondary metabolite content, antioxidant enzyme activity, and mitigated chilling injury in *Punica granatum* L. In another study, loquat fruit were treatment with MeJA under cold stress. MeJA treatment effectively reduced harmful effect of chilling injury and enhanced antioxidant enzymes system (Jin et al., 2014). Under salinity stress, JA effectively improved growth characteristics, proline, metabolite content, and reduced oxidative damage in bitter melon (Alisof et al., 2020). Under cadmium toxicity, low MeJA significantly lowered MDA levels while increasing root development, antioxidant enzyme defense system, and chlorophyll content in *Capsicum frutescens* L. (Yan et al., 2013). Faghih et al. (2017) reported considerable increments in antioxidant activity by pretreatment of MeJA under salt stress in strawberry seedlings.

**Table 2:** Phytohormones enhanced abiotic stress tolerance of horticultural crops.

Plant hormone	Plant name	Stress type						Reference
		Drought	Salinity	Heat	Cold	Acid rain	Heavy metal	
Brassinosteroids	Radish						X	Choudhary et al., 2012
Melatonin	Fenugreek	X						Zamani et al., 2019
Jasmonic acid	Peach				X			Zhao et al., 2021
Salicylic acid	Tomato			X				Jahan et al., 2019
GABA	Peach				X			Yang et al., 2011
Brassinosteroids	Pepper	X						Hu et al., 2013
Salicylic acid	Bitter melon		X					Alisof et al., 2020
Jasmonic acid	Tomato						X	Zhao et al., 2016
Strigolactones	Rose			X				Djennane et al., 2014
Brassinosteroids	Lettuce		X					Serna et al., 2015
Jasmonic acid	Strawberry	X						Yosefi et al., 2020
GABA	Tomato				X			Malekzadeh et al., 2014
Strigolactones	Pea						X	Cooper et al., 2018
Melatonin	Tomato					X		Debnath et al., 2020
Jasmonic acid	Okra		X					Azooz et al., 2015
Salicylic acid	Banana				X			Kang et al., 2003
GABA	Melon		X					Xiang et al., 2016
Brassinosteroids	Pea				X			Shahid et al., 2011
Jasmonic acid	Banana			X				Zhao et al., 2013
Strigolactones	Grapevine	X						Min et al., 2019
Melatonin	Cucumber				X			Zhao et al., 2017
Salicylic acid	Peppermint						X	Ahmad et al., 2018
Melatonin	Cucumber		X					Zhang et al., 2020
Brassinosteroids	Cucumber			X				Wei et al., 2015
Strigolactones	Tomato	X						Visentin et al., 2016
Jasmonic acid	Sugar beat	X						Ghafari et al., 2020

Previous literature revealed under abiotic stress environment, JA improved growth status in Solanum nigrum (Yan et al., 2015), increased seed germination in okra (Azooz et al., 2015), enhanced pigments content in strawberries (Yosefi et al., 2020), enhanced leaf gas exchange parameters (Choudhary and Agrawal, 2014) increased antioxidant defense system in cauliflower (Wu et al., 2012), increased osmolytes content in tomato (Bali et al., 2018), reduced ROS production in *Malus* crabapple (Qiu et al., 2019), and reduced heavy metal accumulation in horticultural plants (Dar et al., 2015; Zhao et al., 2016). Amid jasmonates, JA is found notably well known, best characterized and most abundant one. The defense mechanisms of horticultural crops are well-regulated by JA, when exposed to abiotic stresses including drought (Ghafari et al., 2020), salinity (Abouelsaad and Renault, 2018), cold (Zhao et al., 2013), alkalinity (Ge et al., 2010), heat (Chen et al., 2006), and metal stress (Bali et al., 2019). Thus, the JA is capable of reducing various environmental stress vulnerabilities (Raza et al., 2021). Furthermore, exogenous MeJA helped to increase endogenous levels of JA in pea crop (Shahzad et al., 2015). Meng et al. (2009) indicated that MeJA treatment reduced phenolic content while increasing antioxidant enzyme activity due to chilling damage index. In citrus, JA treatment significantly boosted proline content and antioxidant enzyme activity while decreasing ROS-induced oxidative damage (Habibi et al., 2019). Ouli-Jun et al. (2017) reported that the metabolism, root respiration, antioxidant enzymes activity, and osmolytes content were significantly enhanced, whereas, the hydroxyl free radical’s accumulation, MDA and EL content were declined through the exogenous application of MeJA under waterlogging stress in pepper plants. Hence, the adaption of horticultural crops in abiotic stress conditions is positively regulated by JAs.

## Strigolactones

Strigolactones (SLs) are newly discovered multifunctional carotenoid derivative compounds of the plant hormone (Raza et al., 2021). SLs positively regulates seedling growth, photosynthetic efficiency, leaf senescence, blooming, and ion homeostasis (Banerjee and Roychoudhury, 2018). Exogenous supplementation of SLs significantly increased growth, relative water content, anti-oxidant enzyme activity, gas exchange parameters, chlorophyll fluorescence elements and contents in *Vitis vinifera* under drought conditions, while drastically lowering the oxidative injury, stomatal opening, and EL level (Min et al., 2019). Furthermore, the application of SLs activates the transcription of genes related to SLs production. SLs production promotes root architecture and arbuscular mycorrhizal fungus symbiosis, which boosts nutrient intake (Bhoi et al., 2021). Exogenous SLs modified root growth pattern of tomato (Santoro et al., 2020). Similarly, SL applied to apple plants exogenously exhibited significant increases in chlorophyll content and seedlings’ net photosynthetic rate, when exposed to potassium chloride (KCL) toxicity (Zheng et al., 2021). Furthermore, under KCL toxicity, SLs application enhanced POD and SOD activity while decreasing oxidative stress by increasing accumulation of proline, sustaining absorption of mineral nutrient and osmotic equilibrium. The SLs applied exogenously under salt stress significantly increased cucumbers seedlings’ leaf photosynthetic capability, AsA-GSH pool, and decreased oxidative damage (Zhang et al., 2015). Under drought, SLs treatment considerably improved stomata’s sensitivity in tomato (Visentin et al., 2016).

Additionally, the stress tolerance of various horticultural crops was enhanced under abiotic stress conditions by endogenous SLs, which label them as endogenous growth regulators (Ha et al., 2014; Sytar et al., 2019). Banerjee et al. (2017) stated that SLs have received a significant attention during recent years due to their crucial roles in regulation of multiple processes, both physiological and molecular, throughout the responses of plants towards abiotic stresses. SLs primarily functions as a second messenger in shoot branching by dampening auxin transport in the stem and so suppressing axillary bud development (Saeed et al., 2017). Furthermore, in low-light conditions, SLs foliar spray improved tomato growth traits, chlorophyll fluorescence parameters, pigment molecule and photosynthetic assimilation. Furthermore, SLs application increased antioxidant enzyme gene expression while decreasing H2O2 and MDA levels in tomato under low light-stress (Lu et al., 2019). Exogenous application of SLs in peas significantly increased photosynthetic pigment and shoot branching under cold stress (Cooper et al., 2018). Exogenous SLs improved salt stress tolerance in *Solanum lycopersicum* L. by boosting antioxidant defense mechanism, chlorophyll and carotenoids content, and endogenous SLs synthesis (Liu et al., 2022). Seed priming with SLs in Lupine resulted in improved germination of seeds and growth of seedlings, and helped to enhance proline content and decline MDA content. Additionally, the antioxidant enzymes activity and glyoxalase system of lupine seedlings showed improvements after application of SLs (Omoarelojie et al., 2020). SLs supplementation regulating antioxidant defense system, increasing nutrient uptake, and reduced MDA accumulation in cucumber under salt stress (Zhang et al., 2022). Under cadmium toxicity, SLs significantly reduced MDA accumulation and enhanced root vigor, activity of antioxidant enzymes, and flavonoid biosynthesis by modulating there encoding gene in melon seedlings (Chen et al., 2022). Overall, SLs shows a vital function in the abiotic stress physiology of horticultural crops in response to various environmental stresses.

## GABA

One of the non- proteinogenic amino acid in plants is the Gamma-aminobutyric acid (GABA), which acts as a signaling molecule, having well-recognized ubiquitous status and multifaceted roles (Ansari et al., 2021). In horticultural crops, GABA is known to mediate various physiological function such as regulation of seed germination (Kumar et al., 2019), osmolyte accumulation (Li et al., 2021), balanced photosynthetic capacity (Wu et al., 2020), root architecture (Seifkalhor et al., 2019), plant yield regulation (Sita and Kumar, 2020), and ion homeostasis (Fait et al., 2008), redox homeostasis (Ansari et al., 2021), and antioxidant defense system (Hasan et al., 2021). The morphological and physiological functions of plants including production of proline, soluble sugar, and polyamine metabolism, as well as photosynthetic efficiency, are reported to be significantly improved by the application of GABA under abiotic stress (Jalil and Ansari, 2020). Chen et al. (2018) stated that this molecule is also known for mitigating excess ROS formation under stress conditions, mainly through activation of antioxidant defense mechanism. Furthermore, exogenous GABA supplementation markedly enhanced leaf photosynthesis traits, modulate stomatal opening, improved root growth, balanced ion homeostasis, enhanced osmolytes accumulation, and stress related protein in black pepper under PEG-induced stress (Vijayakumari and Puthur, 2016). Malekzadeh et al. (2013) revealed that foliar application of GABA efficiently improved seedling growth status, proline content, leaf water potential, stomatal opening, and antioxidant enzymes activity in tomato seedling under cold stress. According to Su et al. (2019), GABA shunt components are essential for ion homoeostasis. Under cold stress, GABA treatment significantly increased biomass yield, chlorophyll content, antioxidant enzymes, and lowered ROS production and cell membranes integrity in peaches (Shang et al., 2011). GABA is the primary mediator of induced leaf senesces under oxidative stress. Under short/low light stress, GABA increased chilies stress resistance through activation of antioxidant defense system, and increasing photochemical efficiency. Furthermore, Li et al. (2017b) described that GABA treatment increased gas exchange traits, chlorophyll content and fluorescence characteristics, SOD and CAT activity, and decreased levels of MDA content in chilies.

During environment stress such as salt stress, drought, heavy metals, low and high temperature stresses (Sita and Kumar, 2020; Ansari et al., 2021; Hasan et al., 2021), the production of GABA is escalated with such an intensity that exceeds the cellular levels of this non-proteinogenic amino acid than those amino acids having role in protein synthesis (Srivastava et al., 2021). Notably, the production of high amounts of GABA were observed under abiotic stress conditions, and there found a nexus between its metabolism and other factors including defense against oxidative stress, antioxidant enzymes’ upregulation, osmolytes regulation, and balance ion homeostasis (Khan et al., 2021). The abiotic stress resistance of plants has been successfully improved through GABA priming. The growth of muskmelon was also significantly increased with application of GABA under calcium nitrate stress. Furthermore, GABA treatment significantly increased ADC, PAO, OCD, DAO and SMDC activity. Exogenous GABA treatment successfully increased spermidine and spermine levels while decreasing level of putrescine in leaves, enhancing polyamine biosynthetic concentration (Hu et al., 2015). Furthermore, exogenous application in carrots, tomato, and peach increases endogenous GABA levels, resulting in increased enzymatic activity, pigment content, and ultimately resistance of plants against abiotic stress (Yang et al., 2011; Koike et al., 2013; Bashir et al., 2021). The oxidative stress recovery is also linked with GABA application, which helps to scavenge the excess ROS production through a disturbance in intracellular redox

## Future outlook

The adversities linked with abiotic stress factors are surging day-by-day, gaining attention of scientific research from plant biologists so as to avoid threats to sustainable agricultural production I future. Phytohormones have emerged as a viable technique in current stress management because they protect plants from numerous abiotic stressors by boosting antioxidant enzyme activity, lowering oxidative damage, and promoting plant development (**Figure 3**). Therefore, in flood-, drought-, and saline-prone areas worldwide, the sustainability of crops production can be maintained through utilization of phytohormones, which are proved to better the abiotic stress resistance of horticultural crops. Along with improving the stress resistance in abiotic stresses, the application of phytohormones is also known for ensuring the harmonization of germination process, mainly by increasing the viability and breaking the seed dormancy. Current review is an attempt to provide useful insights into the exogenous application of phytohormones and the role played by these in developing and enhancing the plants’ defense mechanisms. In recent two decades, the identification and characterization of metabolizing enzymes related to phytohormones has been focused by the researchers. Further, for better understanding of growth-regulation mechanisms induced by phytohormones and bringing more clarity to elusive interactive events, intensive executions of phytohormones crosstalk research is being made recently. Finally, in future research, the aim of modernizing agricultural production with the engineering of abiotic stress-resistant crops can be aligned with the manipulation of phytohormones level and their subsequent action at pertinent developmental stage in appropriate tissue/organ.

## Author contributions

YZ and YH: Conceptualization, Literature survey, Writing major original draft, Review structure. XW and XC: Literature survey, Writing- review and editing, Figure designing. KW: Literature survey, Writing- review and editing. YW: Reviewing and editing, References collection. YH: Supervision. All authors contributed to the article and approved the submitted version.
